# Connection-type-specific biases make uniform random network models consistent with cortical recordings

**DOI:** 10.1152/jn.00629.2013

**Published:** 2014-06-18

**Authors:** Christian Tomm, Michael Avermann, Carl Petersen, Wulfram Gerstner, Tim P. Vogels

**Affiliations:** ^1^School of Computer and Communication Sciences and School of Life Sciences, Brain Mind Institute, École Polytechnique Fédérale de Lausanne, Lausanne, Switzerland;; ^2^Laboratory of Sensory Processing, Brain Mind Institute, Ecole Polytechnique Federale de Lausanne, Lausanne, Switzerland; and; ^3^Centre for Neural Circuits and Behaviour, Department of Anatomy, Physiology and Genetics, The University of Oxford, Oxford, United Kingdom

**Keywords:** neuronal network models, random connectivity, layer 2/3 sensory cortex

## Abstract

Uniform random sparse network architectures are ubiquitous in computational neuroscience, but the implicit hypothesis that they are a good representation of real neuronal networks has been met with skepticism. Here we used two experimental data sets, a study of triplet connectivity statistics and a data set measuring neuronal responses to channelrhodopsin stimuli, to evaluate the fidelity of thousands of model networks. Network architectures comprised three neuron types (excitatory, fast spiking, and nonfast spiking inhibitory) and were created from a set of rules that govern the statistics of the resulting connection types. In a high-dimensional parameter scan, we varied the degree distributions (i.e., how many cells each neuron connects with) and the synaptic weight correlations of synapses from or onto the same neuron. These variations converted initially uniform random and homogeneously connected networks, in which every neuron sent and received equal numbers of synapses with equal synaptic strength distributions, to highly heterogeneous networks in which the number of synapses per neuron, as well as average synaptic strength of synapses from or to a neuron were variable. By evaluating the impact of each variable on the network structure and dynamics, and their similarity to the experimental data, we could falsify the uniform random sparse connectivity hypothesis for 7 of 36 connectivity parameters, but we also confirmed the hypothesis in 8 cases. Twenty-one parameters had no substantial impact on the results of the test protocols we used.

the simplicity of their implementation as much as the lack of experimentally unambiguous data makes networks of uniform random and sparsely connected neurons a popular model to capture the dynamics of real nervous systems (Van Vreeswijk and Sompolinsky 1996; Amit and Brunel 1997; Brunel 2000; Vogels and Abbott 2005; Destexhe and Contreras 2006; Morrison et al. 2007; Hertz 2010; Renart et al. 2010). Connection probabilities for uniform random networks can be obtained from pairwise recordings (Markram et al. 1997; Feldmeyer et al. 1999; Holmgren et al. 2003; Feldmeyer et al. 2006; Thomson and Lamy 2007; Helmstaedter et al. 2008; Lefort et al. 2009), but pairwise statistics can be afflicted with artifacts from slicing injuries (Song et al. 2005) and provide only limited possibilities to extract the absolute number of synapses per neuron (we call this value the neuron's “in- or out-degree,” depending on whether we count incoming or outgoing connections). More importantly, small experimental data sets make it impossible to estimate degree distributions, so that we cannot know about the potentially convergent (i.e., many neurons connect to only a few) or divergent (i.e., few neurons connect to many) nature of specific connection types.

The analysis of recordings with multiple electrodes (Song et al. 2005; Perin et al. 2011) shows that specific connectivity patterns, known as motifs, are more frequent than predicted by uniform random network architectures. These properties have been thought to be reminiscent of so-called small-world architectures (Watts and Strogatz 1998) and scale-free network models (Barabási and Albert 1999). Small-world models introduce a geography in which neighboring cells are connected more densely and distant nodes are connected sparsely. Scale-free networks also produce nonuniform-random architectures that are characterized by highly connected hub neurons embedded in a sparser background connectivity. Both of these types of models have been fitted to the experimental data with some success (Feldt et al. 2011; Prettejohn et al. 2011; Bonifazi et al. 2009). However, the experimental data are limited by the number of electrodes used, and even with 12 simultaneous electrodes only patterns with up to 8 cells can be classified to the point of statistical significance (Perin et al. 2011).

Optical identification and stimulation techniques such as fluorescent labeling of cells, glutamate uncaging, and channelrhodopsin stimulation (Deisseroth et al. 2006; Miesenböck 2011; Cardin 2012; Packer et al. 2013) make it feasible to collect data from a larger number of neurons. Thus more complex connectivity patterns, especially in regard to cell-type specific connectivity rules (Yoshimura and Callaway 2005; Yoshimura et al. 2005; Fino and Yuste 2011; Bock et al. 2011; Avermann et al. 2012; cf. Table 1), become apparent. These findings complement the results from intracellular recordings and show that the connectivity between neurons can be very specific. However, conclusions about the overall structure of the network are still lacking, because neither all inputs or outputs of a cell nor all cells can be counted in their entirety. The in- and out-degree distributions of a neuron type (adjusted by the parameters d_in_ and d_out_, respectively, see methods), i.e., the distribution of the number of cell-type-specific synapses neurons make or receive, or, in other words, how heterogeneously a network is connected, is thus still unknown. For the same reason, it is impossible to evaluate cell-type-specific inhomogeneties in synaptic weight distributions: from the current data sets we cannot evaluate if neurons connect with similar or widely different average synaptic weights. In fact, the challenges such studies face [simultaneous (intracellular) recording and consecutive stimulation of many thousand neurons] are probably going to make such data sets difficult to obtain for the foreseeable future. These technical limitations have ensured that uniform random network connectivity is still the de facto working hypothesis for many simulations.

**Table 1.** Probabilities of sharing inputs

	x	Δexp	→	Δexp	←	Δexp	↔	Δexp
E → E								
Experiment	0.038	0.0			0.201			0.0
Uniform random model	0.059	0.021			0.059			0.142
Best model	0.078	0.040			0.173			0.028
E → FS								
Experiment	0.051	0.0	—	—	0.013	0.0	0.172	0.0
Uniform random model	0.098	0.047	—	—	0.098	0.085	0.098	0.074
Best model	0.027	0.024	—	—	0.076	0.063	0.151	0.021
E → NFS								
Experiment	0.040	0.0	0.030	0.0	0.050	0.0	0.050	0.0
Uniform random model	0.081	0.041	0.081	0.051	0.081	0.031	0.081	0.031
Best model	0.066	0.026	0.070	0.040	0.069	0.019	0.067	0.017

Probabilities of sharing input for the structurally adjusted network with all significant parameters (model) and the corresponding experimental findings. Shown are the probabilities for all tested pairings. Neurons were either unconnected (x), unidirectionally connected (→, ←), or bidirectionally connected (↕). The experimental results of Yoshimura et al. (2005) only differentiated connected and unconnected for pairs of excitatory neurons. Hence, only 1 value is shown. Yoshimura and Callaway (2005) only analyzed unidirectional connections from FS interneurons to excitatory cells. The probability of shared input for the unidirectional E → FS connection remained untested.

Here, instead of trying to untangle the connectivity of cortical networks experimentally, we generated thousands of network architectures and asked which ones produced plausible results, i.e., which networks produced similar triplet (structural) statistics as observed in layer 2/3 of rat visual cortex slices by Yoshimura and Callaway (2005) and Yoshimura et al. (2005), and subthreshold responses similar to those in layer 2/3 mouse barrel cortex slices shown in Avermann et al. (2012). We obeyed global pairwise connectivity statistics but deviated from uniform random connectivity by introducing parameters that created both structural and synaptic weight inhomogeneities (see Fig. 1., cf. Roxin 2011; Koulakov et al. 2009; Pernice et al. 2013). We did not consider distance-dependent connection probabilities because the experimental evidence for the spatial scale of our models (ca. 3 × 10^−3^ mm^3^) is inconclusive: on this scale all possibilities, no distance (Lefort et al. 2009; Avermann et al. 2012), weak (Packer and Yuste 2011), and even strong (Holmgren et al. 2003) distance dependency, have been observed.

To investigate the architecture of each generated network, we used similar analysis methods as in previous experimental investigations: to compare the connectivity structure, we calculated the connection probabilities of randomly drawn neuron triplets, as reported by Yoshimura and Callaway (2005) and Yoshimura et al. (2005). To investigate the biological plausibility of synaptic weight distributions we used the following proxy: we mimicked recent experimental work (Avermann et al. 2012) in which a small number of channelrhodopsin expressing pyramidal neurons of layer 2/3 neurons in mouse barrel cortex were light activated to emit single spikes. The maximum voltage deflection of the postsynaptic response was measured in nonexpressing excitatory neurons, as well as in fast-spiking and nonfast-spiking GABAergic neurons and cataloged in maximum response histograms (MRH). We used the same protocol in our networks and could thus compare the resulting MRHs to identify important architectural qualities that increased the similarity score between experiment and model.

The networks we investigated were composed of excitatory (E), fast-spiking (FS), and nonfast-spiking (NFS) neurons. We could change the statistics of each of the nine resulting connection types independently with two structural and two weight manipulations. To investigate this 36-dimensional parameter space we simplified our approach as follows (see Fig. 2): for each parameter we chose only two possible values. Additionally, we divided our parameter exploration into four steps. First, we investigated (in 1,024 models) the impact of experimentally available structural parameters. In a second step (and a new set of 1,024 models with appropriately adjusted structural parameters), we investigated the impact of correlations in the synaptic weights on the spiking responses as described above. In a third step and fourth step (with 64 and 1,024 models, respectively), we investigated the impact of experimentally nonverifiable dimensions, e.g., the impact of the weight correlations of outgoing synapses of NFS inhibitory neurons. We sorted and visualized the results of the parameter scans through clutter-based dimensional reordering (CBDR; also known as “dimensional stacking,” LeBlanc et al. 1990; Peng 2005; Taylor et al. 2006), a visualization technique that preserves the original dimensionality of the data set but is otherwise similar to principle component analysis. With the help of CBDR we identified which of the parameters significantly changed the network constitution and its similarity to the described biological data and were thus “crucial” for an accurate reproduction of neuronal networks. The required adjustments were highly connection specific. For example, recurrent E → E connections required a deviation from the uniform random network architecture both in the connection structure (in- and out-degree distributions) and in the input weight correlations. E → NFS connections, on the other hand, had to be connected uniformly randomly to produce the best results. In the following (cf. Table 2), we discuss which out of the 36 tested connection parameters must deviate from uniform random connectivity (7/36) in their structural and weight statistics to reproduce the datasets we used, which parameters must obey uniform random connectivity statistics (8/36), and which are not crucial (21/36) (and partially not appropriately testable due to lack of structural data). We show that these conceptually simple changes created networks that produced biologically realistic dynamics with high degree of similarity to experimental data. The improved network architectures are available via ModelDB (http://senselab.med.yale.edu/modeldb/ShowModel.asp?model=156040).

**Table 2.** Summary of parameter effects

	d		ς	
	In	Out	In	Out
E → E	*XXX*	*XXX*	*XXX*	✓✓✓
E → FS	*XXX*	✓✓✓	*XXX*	*X*
E → NFS	(✓✓)	✓✓✓	*XXX*	✓
FS → E	—	—	—	—
FS → FS	—	(✓✓✓)	—	(✓✓)
FS → NFS	—	—	—	—
NFS → E	✓✓✓	—	—	—
NFS → FS	—	—	—	—
NFS → NFS	—	—	—	—

Parameters that significantly altered similarity scores between model and experimental data are listed with a check mark indicating compliance with the classical random network approach and a crossout for cases in which the random connectivity hypothesis was falsified. Significance levels were 0.1, 0.05, and 0.01, marked by 1, 2, or 3 check marks or crosses, respectively Noncrucial parameters are marked with “—”. Parameters that failed their Bonferroni confirmation are displayed in ().

E, excitatory; FS, fast spiking; NFS, nonfast spiking; d, degree distribution.

## METHODS

We simulated networks of excitatory and inhibitory fast-spiking and nonfast-spiking neurons with population sizes that mirrored cell counts of Lefort et al. (2009) in mouse barrel cortex (E: 1691, FS: 97, and NFS: 133). All simulations utilized the NEST simulator (Gewaltig and Diesmann 2007) and pyNN (Davison et al. 2009).

### 

#### Neuron model.

Single neuron dynamics were simulated using the Adex integrate-and-fire neuron models as provided with the NEST simulator (Brette and Gerstner 2005; Gewaltig and Diesmann 2007). Closely following Brette and Gerstner (2005), we use an integrate-and-fire model with adaptation defined by *CV̇* = *f*(*V*) − *w* + *I*, in which *C* is the membrane capacitance, *f*(*V*) is a function describing the passive properties and the spiking mechanism, *w* is an adaptation variable, and *I* is the synaptic current. The intrinsic parameters of the model relevant for *f*(*V*) (membrane capacitance, membrane time constant, resting membrane potential, reset potential, threshold, slope factor, refractory time constant, adaptation parameters, and adaptation time constant) were taken from previous fits to experimental recordings of mouse barrel cortex (see Table 3, Mensi et al. 2012). The (excitatory and inhibitory) synaptic time constants and the reversal potentials (0 and −75 mV for excitation and inhibition, respectively) were fitted directly to available experimental data (Avermann et al. 2012). To introduce heterogeneity in the network populations, we used multiple parameter sets for each cell type (9 E, 9 FS, and 8 NFS). For each cell of the network one of these parameter sets was chosen at random, so that any given network consisted of multiples of 26 unique model neurons. This heterogeneity was introduced for biological plausibility but did not alter the final results.

**Table 3.** Single neuron parameters

Label	cm	tau m	v rest	v reset	tau refrac	v thresh	delta T	a	b	tau w	tau syn I	tau syn E	e rev I
E1	0.08	22.44	−67.00	−36.74	4.00	−43.08	0.50	0.00	19.39	89.00	15.00	4.00	−75.00
E2	0.05	9.05	−75.81	−37.59	4.00	−45.09	0.50	0.00	22.46	55.58	15.00	4.00	−75.00
E3	0.05	8.31	−65.71	−28.44	4.00	−37.80	1.30	0.00	14.43	45.66	15.00	4.00	−75.00
E4	0.08	22.44	−67.00	−36.74	4.00	−44.22	0.50	0.00	19.39	89.00	15.00	4.00	−75.00
E5	0.05	9.05	−75.80	−37.59	4.00	−49.66	0.50	0.00	22.46	55.58	15.00	4.00	−75.00
E6	0.08	33.32	−69.93	−34.75	4.00	−35.18	1.10	0.00	28.01	113.09	15.00	4.00	−75.00
E7	0.05	6.13	−67.83	−39.37	4.00	−45.37	0.60	0.00	11.31	61.07	15.00	4.00	−75.00
E8	0.06	7.77	−58.59	−31.94	4.00	−39.76	0.50	0.00	16.86	76.12	15.00	4.00	−75.00
E9	0.09	12.10	−71.98	−36.89	4.00	−42.91	0.70	0.00	15.84	71.76	15.00	4.00	−75.00
FS1	0.05	8.60	−72.13	−56.11	4.00	−46.89	0.50	0.00	3.13	20.01	4.00	2.25	−75.00
FS2	0.06	8.47	−70.12	−60.11	4.00	−47.10	0.40	0.00	5.16	32.02	4.00	2.25	−75.00
FS3	0.05	3.65	−62.71	−53.61	4.00	−40.86	0.50	0.00	0.01	0.10	4.00	2.25	−75.00
FS4	0.04	5.40	−66.29	−51.88	4.00	−39.37	2.20	0.00	4.02	21.15	4.00	2.25	−75.00
FS5	0.04	6.43	−67.81	−45.31	4.00	−39.38	0.40	0.00	6.43	38.46	4.00	2.25	−75.00
FS6	0.05	4.48	−72.04	−56.23	4.00	−43.84	1.10	0.00	5.71	17.79	4.00	2.25	−75.00
FS7	0.05	8.72	−72.38	−55.59	4.00	−47.27	0.50	0.00	2.95	20.11	4.00	2.25	−75.00
FS8	0.06	8.52	−70.04	−60.11	4.00	−46.75	0.50	0.00	5.21	31.85	4.00	2.25	−75.00
FS9	0.05	4.48	−72.04	−56.23	4.00	−43.18	0.50	0.00	5.71	17.79	4.00	2.25	−75.00
NFS1	0.05	6.97	−71.20	−48.42	4.00	−43.46	0.50	0.00	5.82	35.08	5.00	3.25	−75.00
NFS2	0.03	5.66	−63.02	−42.27	4.00	−36.61	0.40	0.00	7.20	75.68	5.00	3.25	−75.00
NFS3	0.04	14.35	−61.65	−40.25	4.00	−40.14	0.60	0.00	10.56	105.28	5.00	3.25	−75.00
NFS4	0.05	6.93	−71.21	−48.42	4.00	−44.47	0.50	0.00	6.00	33.90	5.00	3.25	−75.00
NFS5	0.05	9.93	−59.79	−38.67	4.00	−33.09	0.50	0.00	7.75	56.52	5.00	3.25	−75.00
NFS6	0.04	6.43	−67.81	−45.31	4.00	−39.77	1.10	0.00	6.43	38.46	5.00	3.25	−75.00
NFS7	0.03	5.66	−63.02	−42.27	4.00	−35.39	0.50	0.00	7.20	75.68	5.00	3.25	−75.00
NFS8	0.04	14.35	−61.65	−40.25	4.00	−39.52	0.80	0.00	10.56	105.28	5.00	3.25	−75.00

List of all used single neuron parameters. The label indicates the neuron type (E, FS, and NFS).

#### Network architecture.

The network comprised two parts: the connectivity matrix and the weight matrix. Both of these matrixes could be manipulated independently to construct networks of various connectivity profiles.

#### Connectivity matrix.

The number of connections *N* between two neuronal populations of size *M*_PRE_ and *M*_POST_ was calculated from *N* = *p* × *M*_PRE_ × *M*_POST_, where *p* is the specific, experimentally measured connection probability between PRE and POST populations. For each possible connection type, we used pairwise probabilities reported by Lefort et al. (2009) and Avermann et al. (2012). To create a connectivity matrix between two neuron populations PRE and POST, we thus drew exactly *N* pairs of presynaptic neuron *j* and postsynaptic neuron *i*. The indexes *j* and *i* were drawn independently from normalized, truncated exponential distributions with probability *P*_PRE_(*j*) and *P*_POST_(*i*), respectively. We designed these probability distributions such that the presynaptic probability follows

(1) $$P_{\text{P}\text{R}\text{E}}\left(j\right)=\frac{1}{C}\;e^{-\frac{j\;{\text{d}}_{\text{o}\text{u}\text{t}}}{M_{\text{P}\text{R}\text{E}}}}$$

where *C* is the appropriate normalizing factor to keep the total number of synapses constant,

(2) $$C=\overset{M_{\text{P}\text{R}\text{E}}}{\underset{j=1}{\sum }}e^{-\frac{j\;{\text{d}}_{\text{o}\text{u}\text{t}}}{M_{\text{P}\text{R}\text{E}}}}.$$

**Fig. 1.**
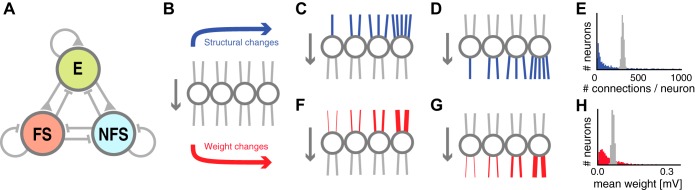
Network and connectivity schematics. *A*: network schematic with excitatory (E), inhibitory fast spiking (FS), and nonfast spiking (NFS) neurons recurrently connected. All 9 connection types of the network could be manipulated individually to divert from uniform, random connectivity (*B*) in which all neurons received and sent near-equal number of synapses (*E*, gray histogram) with near-equal average synaptic weights (*H*, gray histogram). We investigated the effect of altered structural connectivity by changing the in-degree (*C*) and out-degree (*D*) distributions of a connection type so that the number of synapses per neuron was more broadly distributed (*E*, blue histogram) than in a classical random network (*E*, gray histogram). We also investigated the effect of changes in the correlations of incoming (*F*) and outgoing (*G*) synaptic weights of each neuron, so that some neurons received or sent only strong or only weak synapses (*H*, red histogram).

Postsynaptic indexes *i* were drawn independently from an analogous distribution *P*_POST_(*i*), using parameter d_in_ and *M*_POST_. The index lists *i* ∈ {1 . . . *M*_PRE_} and *j* ∈ {1 . . . *M*_POST_} were shuffled so that when X_PRE_ = X_POST_, for X = {E, FS, NFS} and *j* = *i*, neuron *j* ≠ neuron *i*. With d_out_ = 0 or d_in_ = 0 this algorithm generated uniform random connectivity with a low variance in the number of connections per neuron (Fig. 1). Alternatively, with d_in_ > 0 or d_out_ > 0, structured networks with higher variance in the number of connections per neuron were created. In such networks few neurons had many connections and most neurons had very few connections. The resulting distribution is approximately exponential (Fig. 1*E*, blue histogram). For clarity it should be pointed out that d_out_ is not the out-degree, but the parameter used to manipulate the distribution of out-degrees across the network.

We only used two values for d = {0, 5} in our simulations because even a single additional test value increased the number of possible variable combinations by a factor of 50 (to more than 50,000 possible combinations) and consequently increased the computer time to 120 processor days for a single parameter sweep. To insure a sufficient difference between degree distributions, we ran test simulations with d = {1 . . . 15} (data not shown, but cf. Fig. 8*B*). We chose d = 5 as a compromise that created a wide range of different connection numbers per neuron without creating many neurons that were connected to every other neuron in the network. For clarity, we labeled d with a tag of the specific connection type and called it the degree distribution parameter because its value controls the shape of the synapse number distributions. For example, d_out_^FS→E^ was the parameter that controlled the shape of the out-degree distribution of fast-spiking (FS) to excitatory (E) connections.

#### Weight matrix.

For each existing connection, the synaptic strength *w* was drawn from a lognormal distribution,

(3) $$\text{P}\left(w_{ij}\right)=\frac{1}{w_{ij}\sigma \sqrt{2\pi }}\;e^{-\frac{{\left[\text{I}\text{n}\left(w_{ij}\right)-\mu \right]}^{2}}{2\sigma^{2}}}$$

with scale μ and shape σ, chosen so as to maximize the likelihood of and thus to emulate the experimentally measured synaptic strength distributions for each connection type (Lefort et al. 2009; Avermann et al. 2012; Table 4), that is

(4) $$\mu =\frac{1}{N}\sum_{q=1}^{N}\text{I}\text{n}\left(x_{q}\right)$$

(5) $$\sigma^{2}=\frac{1}{N}\sum_{q=1}^{\text{N}}{\left[\text{I}\text{n}\left(x_{q}\right)-\mu \right]}^{2}$$

**Table 4.** Global weight distribution parameters

Connection	μ	σ^2^
E-E	−9.57	0.96
E-FS	−8.56	0.53
E-NFS	−9.94	0.78
FS-E	−9.29	0.83
FS-FS	−8.72	0.32
FS-NFS	−8.17	0.75
NFS-E	−9.36	0.77
NFS-FS	−9.27	0.36
NFS-NFS	−10.07	0.02

Parameters of the lognormal distribution used to determine synaptic weights for all connections.

where *x*_*q*_ are the experimentally measured synaptic strengths and *N* the number of data points. These global weight distributions were subsequently used for all network architectures.

#### Weight correlations.

Additionally, we created heterogeneity in the weight structure of the networks by introducing correlations between the strengths of all incoming or outgoing synapses of the same neuron. To introduce these changes in the weight correlations of single neurons, we drew two sets of scaling values, α_pre_^*j*^ and α_post_^*i*^ (Koulakov et al. 2009) from log normal distributions,

(6) $$P\left(\alpha_{\gamma }\right)=\frac{1}{\alpha_{\gamma }\varsigma \sqrt{2\pi }}\;e^{-\frac{{\left[\text{I}\text{n}\left(\alpha_{\gamma }\right)-\mu_{\gamma }\right]}^{2}}{2\varsigma_{\gamma }^{2}}}$$

where γ stands for pre or post. To parameterize the induction of correlations we set ς_γ_ = {0, 1}, and adjusted the scale μ_γ_,

(7) $$\mu_{\gamma }=-\left(\varsigma_{\gamma }^{2}\right)/2$$

[i.e., μ_γ_^(ςγ=0)^ = 0, and μ_γ_^(ςγ=1)^ = −0.5] to ensure that the mean of the distribution was equal to 1, thus leaving the global weight distributions undisturbed. We then multiplied the original weights *w*_*ij*_ with α_PRE_^*j*^ of the presynaptic partner and α_POST_^*i*^ of the postsynaptic partner. For ς = 0 the distribution of *P*(α_γ_) collapsed to a δ-function around 1. No weight correlations were induced, leading to a narrow distribution of average weights per neuron (Fig. 1*H*, gray). For ς = 1, the distribution of values in α_γ_ forces (row and/or columnwise) correlations of the synaptic weights of each neuron, leading to increased variance and skewness of the distribution of average weights (Fig. 1*H*, red). For similar reasons as mentioned in the case of parameters *d*_*x*_, we restricted ourselves to only two parameter values (data not shown but cf. Fig. 8*C*). We labeled ς depending on the locus of the pre- or postsynaptic weight correlations as ς_out_ or ς_in_, respectively. Additionally we added a tag to identify the connection type we manipulated. For example, ς_in_^E→NFS^ in controls the input weight correlations in the E → NFS connection.

#### Structural similarity to experimental data.

By definition, the networks we constructed exhibited the same pairwise connectivity as biological networks reported in Lefort et al. (2009) and Avermann et al. (2012). To investigate the occurrence of more complex patterns in the connectivity matrix, we compared them to some of the most complete studies of connectivity profiles between excitatory and inhibitory neurons (Yoshimura and Callaway 2005; Yoshimura et al. 2005). In these publications, pairs of neurons in cortical layer 2/3 of rat visual cortex were examined for common excitatory input from the same layer. Their cell type (E, FS, or NFS) and their connectivity (connected, unconnected, and bidirectionally connected) was cataloged, and the fraction of cells with shared input from a third cell was recorded over many tested pairs according to their cell type and connectivity (cf. Fig. 2 in Yoshimura and Callaway 2005 and Fig. 3 in Yoshimura et al. 2005). This led to a set of probabilities *P*_exp_ that described the expected frequency of triplet motifs with specific cellular compositions in the network. In this data set, one of the recorded neurons was always excitatory, and the second was an E, FS, or NFS neuron, so no data were available for some of the categories (E → FS, FS → FS, FS → NFS, NFS → FS, and NFS → NFS).

To extract the same information from our models, we drew random pairs of neurons for each known category. The number of presynaptic neurons they shared, divided by the total number of inputs, led to the same probability estimate *P*_mod_ for triplet motifs as reported in Yoshimura and Callaway (2005) and Yoshimura et al. (2005).

To measure the similarity of the connection probability *P*_model_ of the model networks and *P*_exp_ of the experimental data, we computed the root mean square error, RMSE = $\sqrt{Q^{-1}\sum_{q=1}^{Q}{\left(P_{\text{e}\text{x}\text{p}.}^{q}-P_{\mathrm{mod}\text{e}\text{l}}^{q}\right)}^{2}}$, between the probabilities *P*_model_ of all *Q* = 9 reported connection categories (cf. Table 1).

#### Test stimulus and response similarity.

To investigate the dynamic behavior of our networks, we emulated recent experiments (Avermann et al. 2012) in which an in vitro optogenetic stimulation protocol was used to evoke spikes in a transfected population of excitatory neurons. The maximum amplitude *x*_*i*_ of the poststimulus subthreshold voltage responses of randomly chosen neurons near the stimulation site were binned into MRH for each cell type γ. Pooled over many trials, these histograms describe the probability *P*_exp_^γ^(*x*_*i*_) with which a maximum response amplitude can be expected to occur. We aimed to mimic these experiments as closely as possible by stimulating 25 random excitatory neurons of our model networks to emit a single spike. We recorded the responses from 10 independently initialized simulations and constructed the corresponding MRH and thus *P*_model_^γ^(*y*_*i*_) for each cell type (see Fig. 5). Varying the number of spike-emitting neurons introduced noisy variations in the shape of the response histograms that could not explain the experimental distributions.

To quantify the similarity of model and experimental response distributions, we calculated for each neuron type γ (E, FS, or NFS) the log-probability that the experimental response ${\hat{x}}_{i}$ could have been model response ${\hat{y}}_{i}$. Assuming statistical independence between trials, this log-likelihood is given by

(8) $$l_{\gamma }=\underset{i}{\sum }\text{log}\;P_{\text{mod}\text{e}\text{l}}(x_{i})$$

Initially we used the sum ℓ = ℓ_E_ + ℓ_FS_ + ℓ_NFS_ over all neuron types as a single value to describe the similarity of the MRHs in the experimental data and in the model. We also used ℓ_E_ and ℓ_FS_ separately (see Fig. 6), and we used ℓ_NFS_ (see Fig. 7).

#### Impact of single parameters.

We used two-tailed Student's *t*-tests to analyze whether a given parameter change (from *d* = 0 to *d* = 5, or from ς = 0 to ς = 1) had a significant effect on the similarity of the network models to the experimental data. For every parameter, we separated the results of the complete parameter sweep into two populations in which this parameter of interest was one of the two possible values. We called a parameter “crucial” if the populations of RMSE scores from these models where different at the <0.05 significance level. In a second iteration, we fixed all crucial parameters at their optimal value and excluded all models with nonoptimal crucial parameters from further analysis. We then reanalyzed the resulting subspace in the same way as above to reveal additional crucial parameters. This procedure was reiterated until no more crucial parameters were found. For the structural analysis (see Fig. 3), we found all but one (d_in_^E→FS^) crucial parameter in the first iteration. Note that *d_in_^E→NFS^ failed Bonferroni confirmation (see explanation below) and was thus marked with an asterisk. For the analysis of *step II* (see Fig. 6), crucial parameters were revealed in the following order: ς_out_^E→FS^, ς_in_^E→E^, and ς_in_^E→FS^ were found to be crucial in the first iteration, and ς_out_^E→E^ in the second. Note that *d_out_^FS→FS^, found in the third, and *ς_out_^FS→FS^, found in the fourth iteration failed Bonferroni confirmation (see below). The crucial parameters found in *step III* (see Fig. 7) were both found in the first iteration of analysis.

**Fig. 2.**
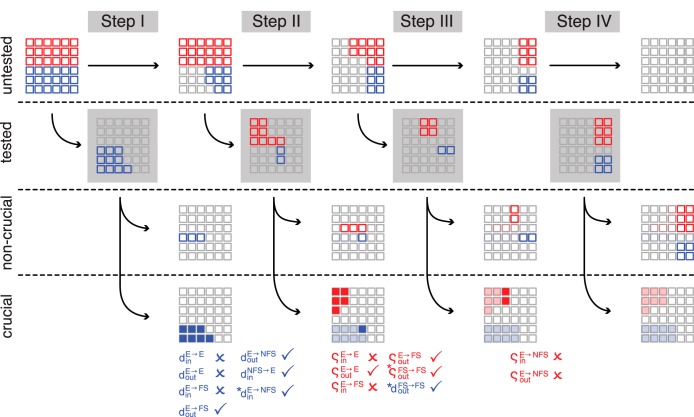
Steps of the analysis. We considered 18 structural and 18 weight parameters in our parameter search, symbolized as blue and red squares, respectively, at *top left*. We proceeded in 4 consecutive analysis steps, shown along the horizontal axis of the schematic. During each step we tested a limited number of parameters, shown in color in the second row, labeled “tested.” Afterwards, we sorted the tested parameters into “noncrucial” and “crucial” (*3rd* and *4th row*, respectively), depending on whether a parameter had a significant effect on the similarity of the model to the experimental data sets. Crucial parameters are listed in the lower margins. Check marks indicate which parameters confirm the random architecture hypothesis, and crosses mark the parameters for which the null hypothesis was falsified. *Step I* comprised 10 structural parameters. *Step II* comprised 2 structural and 8 weight parameters. In *step III* we tested the effect of 2 structural and 4 weight parameters, and in *step IV* we tested the remaining 4 structural and 6 weight parameters. We found 15 crucial parameters, 3 of which failed Bonferroni confirmation (marked with asterisks in the margin).

To ensure statistical rigor, we retested all parameters against Bonferroni-corrected significance values *p*_B_ = α/N, where α = 0.05 is a conservative single trial significance level, and the correction factor *N* = 14, 28, 6 for *steps I*, *II*, and *III*, respectively. The three crucial parameters that failed their Bonferroni confirmation (*d_in_^E→NFS^, *d_out_^FS→FS^, and *ς_out_^FS→FS^) are marked with parentheses in Table 2 and Fig. 2 and asterisks (*) throughout the text. Table 2 summarizes the results of our analysis.

#### High-dimensional parameter analysis.

To visualize the high-dimensional parameter space of our investigation, we used CBDR (LeBlanc et al. 1990; Peng 2005; Taylor et al. 2006), often also referred to as dimensional stacking. This technique stacks high-dimensional result spaces into two-dimensional images by sequentially nesting pairs of parameters into quadrants of the next pair. Through ranking the order of dimensions by how much they affect the result space, and nesting them according to their rank, with the biggest affecters on the outside, CBDR can reveal the underlying structure of the space itself and visualize which dimensions have the biggest impact on the result. To read a CBDR plot, one should first consider the outer dimensions of each plot, because they represent dimension along which the greatest change was observed. The impact of a parameter can thus be also assessed by the level at which it is nested.

#### Reciprocity.

Reciprocity *R* was defined as the probability of two neurons to be connected to each other (Song et al. 2005). In the uniform random case, this can be determined by calculating *R* = *p*^2^ where *p* is the connection probability. In the adjusted networks, we measured reciprocity by simply counting the number of bidirectional connections, and dividing by the number of possible connections, *M*_PRE_ × *M*_POST_, with population sizes *M*_PRE_ and *M*_POST_, respectively. Although Song et al. (2005) and Perin et al. (2011) were able to supply the probabilities for motifs with and even more participant neurons, the sheer magnitude of the combinatorial possibilities in our networks prevented us from looking for these motifs in realistic time frames.

#### Small-world-ness.

We determined the small-world-ness (Watts and Strogatz 1998) of our networks by calculating a small-world-ness value (Humphries and Gurney 2008),

(9) $$S=\frac{C_{\text{t}\text{e}\text{s}\text{t}}}{C_{\text{r}\text{a}\text{n}\text{d}}}*\frac{L_{\text{r}\text{a}\text{n}\text{d}}}{L_{\text{t}\text{e}\text{s}\text{t}}}$$

for each tested network, compared to a uniform random network. Here, *L* is the average shortest path length between any two cells in a network and *C* is the clustering coefficient, determined by dividing the number of existing connections of each neuron by the number of possible connections and averaging over the entire population. A small-world network is characterized by a higher average clustering coefficient and a lower average shortest path length than a uniform random network. Thus networks with *S* > 1 can be considered small-world networks.

## RESULTS

Theoretical work on neuronal networks began long before the publication on motifs and fine structure of their architecture, when all that was known was that their connectivity is sparse. The most parsimonious description of connectivity that only depends on the one known statistic (the sparseness) is an “Erdos-Rényi network” (Erdos and Rényi 1961), i.e., a uniform random network. It has long been suspected that such uniform random network architectures do not accurately describe the architecture of real cortical networks. Here, we tested the uniform random connectivity hypothesis by comparing the structure and the dynamical response properties of thousands of independently generated network architectures with two experimental data sets. We considered a neural network with excitatory (E), as well as fast spiking (FS), and nonfast spiking (NFS) inhibitory neurons. We controlled the statistics of each connection type individually while keeping the overall connectivity constant (see methods) and could thus assess the impact of our statistical manipulations on the similarity of model and experiment (Fig. 1).

We examined the impact of changing the distribution of the number of incoming synaptic connections a given neuron receives (the “distribution of in-degrees,” adjusted by d_in_) and the distribution of the number of outgoing synapses (the “distribution of out-degrees,” adjusted by d_out_). In a uniform random network (d = 0, see methods), these distributions are typically narrow (Fig. 1*E*, gray histogram), so that every neuron sends and receives roughly the same number of synapses. By skewing these degree distributions (d > 0), we could create networks in which some of the neurons received (or sent) a large number of synaptic inputs and others received/sent only few (Fig. 1*E*, blue histogram). Since we controlled the total number of synapses in the network, we guaranteed constant global sparseness for all our models, but let the number of synapses per neuron become heterogeneous, so that the connectivity structure at the cellular level could vary notably between different network models.

We also aimed to examine the shape of local weight correlations in the pre- and postsynaptic populations, ς_in_ and ς_out_. In uniform random networks (ς = 0), there were no correlations between the individual synaptic weights onto or from a specific neuron. We could induce correlations by adjusting all incoming or outgoing synapses of a given neuron by a common scaling factor, drawn independently from a distribution that was normalized to ensure that the global weight distributions were kept equal to those measured by Avermann et al. (2012) (see methods). Some neurons could thus send or receive synapses with a very similar strength that differed notably from the average synaptic weight of all network synapses.

**Fig. 3.**
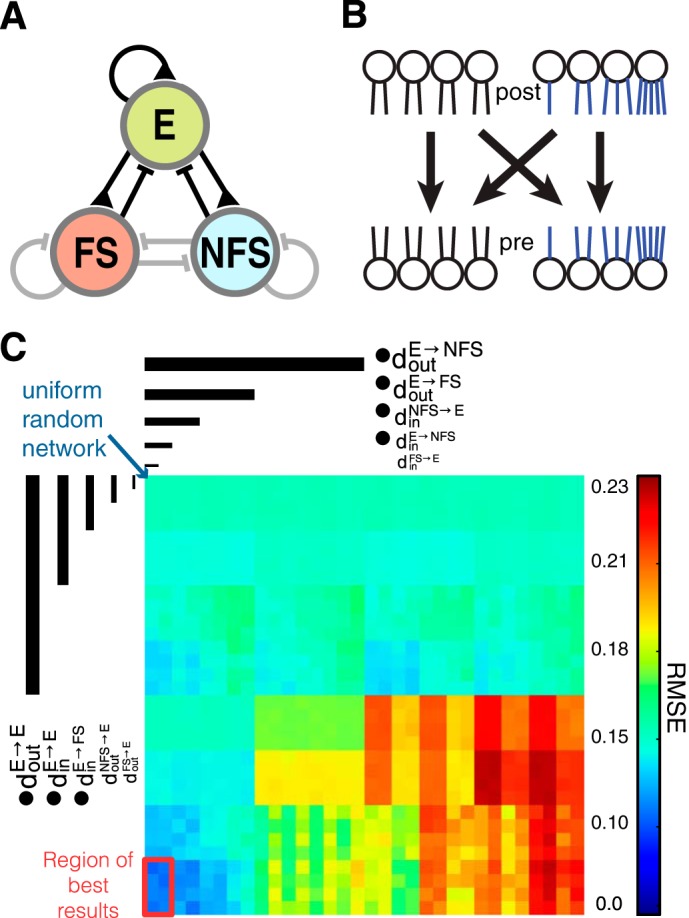
*Step I*: structural parameter exploration. *A*: network diagram as in Fig. 1. Connection types that were investigated in *step I* are shown in black. *B*: schematic of possible connectivity combinations with uniform or skewed in- or out-degree connectivity. *C*: clutter based dimension reordering (CBDR) plot of the RMSE scores (the root mean square error between the observed connection probabilities in experiment and model) of various parameter combinations. CBDR nests parameters into consecutive squares, ranked by their impact on the RMSE value, and helps to visualize important parameters as well as the region of best results (red outline). The blue arrow at *top left* points at the parameter combination that resulted in standard uniform random networks. Crucial parameters are marked with bullet points.

In our networks with three distinct cell populations, and consequently nine unique connection types, these four possible manipulations (d_in_, d_out_, ς_in_, and ς_out_) led to a 36-dimensional parameter space that could not be examined exhaustively by current techniques in reasonable times. Instead, we used a multilayered boot-strap approach (Fig. 2). First, we investigated the variables that affected the structure of the network. In *step I* (Fig. 3), we generated network architectures with one of only two possible degree distributions. Narrow, binomial (d = 0) distributions were used to create networks in which all neurons had similar numbers of incoming or outgoing synapses, and the network's sparseness was thus uniform. Exponentially decaying, skewed (d = 5) in- and out-degree distributions caused some neurons to have many incoming or outgoing synapses, while others had very few. This high variance of the number of connections per neuron created heterogeneous sparsity within the network while global connection probabilities remained unchanged. We examined the effect of changing these parameters for connections E → E, E → FS, E → NFS, NFS → E, FS → E. We excluded all inhibitory to inhibitory connections (FS → FS, FS → NFS, NFS → FS, NFS → NFS) for which we had no experimental data. To compare the resulting architectures to experimental findings, we determined the connection probability of randomly drawn triplets as described in Yoshimura and Callaway (2005) and Yoshimura et al. (2005) and calculated the RMSE between results in the experiment and model (see methods).

Our analysis showed that the uniform random network architecture (Fig. 1*B*) often employed by modeling studies produces an RMSE = 0.07. In other words, uniform random networks on average displayed 7% more or fewer triplet motifs than could be found in experiments, with maximum underestimation of 14% for the occurrence of (at least) monodirectionally connected EEE triplets (Table 1). By changing the in- and out-degree distributions of all mentioned connection types independently, we could observe that the statistics of outgoing E connection caused the biggest changes of the RMSE, but the optimal configuration of parameters was different for each target population (Fig. 3).

For excitatory-excitatory connections (E → E), a high variance of both the in- and the out-degree distributions (d_in_^E→E^ = 5 and d_out_^E→E^ = 5, respectively) produced the best connectivity statistics and reduced the error of EEE triplet motif occurrence by ∼12%. Other changes produced improvements on the order of ∼2%, but the cumulative changes decreased the RMSE between model and experimental data to 0.04. To achieve this, the excitatory to fast-spiking connections (E → FS) required high variance only of the distribution of in-degrees (d_in_^E→FS^ = 5) but a narrow distribution of the out-degrees (d_out_^E→FS^ = 0) connectivity. The connections to NFS neurons (E → NFS) were best described by uniform random connectivity (lower RMSE were achieved with d_out_^E→NFS^ = 0 and *d_in_^E→NFS^ = 0). It should be noted that *d_in_^E→NFS^ failed Bonferroni confirmation (cf. discussion). The only connection from inhibitory cells that had a significant effect on the RMSE was NFS → E, which produced smaller RMSE values when connection numbers were drawn randomly, i.e., from a narrow distribution of synapses per neuron (d_in_^NFS→E^ = 0). The other parameters of the two inhibitory-excitatory connections (and thus d_in_^FS→E^, d_out_^FS→E^, and d_out_^NFS→E^) had virtually no impact on the RMSE value so that several different network architectures produced very similar results (see red box in Fig. 3*C*). The effect of all parameters is summarized in Table 2.

**Fig. 4.**
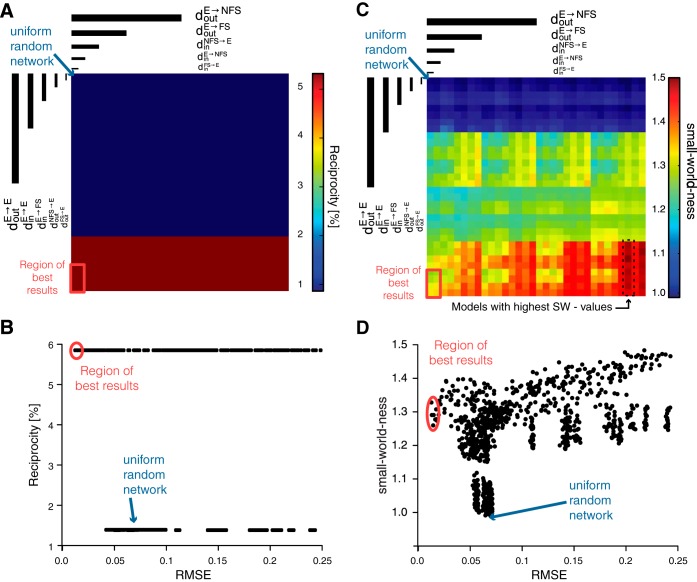
Reciprocity and small-worldness values. *A*: reciprocity values of E → E connections for every parameter combination, plotted with the same dimensional stacking order as in Fig. 3. *B*: scatterplot showing the same network reciprocity values, plotted against their respective RMSE values. *C* and *D*: small-world-ness values for every parameter combination, plotted as in *A* and *B*. In *A*–*D*, the region of best results is outlined in red. The blue arrow shows the uniform random network.

In addition to the comparison to the above data sets by Yoshimura et al. (2005), we also calculated the reciprocity of our model networks (see methods and Fig. 4*A*). We could thus also compare them to the fine structure of 30 experimentally observed networks in layer 5 of acute visual cortical slices from rats aged postnatal day (P)12 to P20 by Song et al. (2005) and in layer 5 of acute somatosensory cortex slices from rats aged P14 to 16 (Perin et al. 2011). Because of the experimentally available data, we focused exclusively on the in- and out degree of E → E connections. When those two crucial parameters were set to their optimal values (d_out_^E→E^ = d_in_^E→E^ = 5), the resulting reciprocity was measured at 0.059, surprisingly similar to the experimentally reported value of 0.054 (Song et al. 2005; Perin et al. 2011). All other combinations of parameter values [d_out_^E→E^ = 0, d_in_^E→E^ = 5], [d_out_^E→E^ = 5, d_in_^E→E^ = 0], and [d_out_^E→E^ = d_in_^E→E^ = 0] resulted in reciprocity values of 0.14, similar to the expected probability of 0.135 calculated from monodirectional connections. Consequently, the RMSE between model networks and the data of Yoshimura et al. (2005) had the smallest values when the reciprocity of the model network was high (Fig. 4*B*).

Cortical networks have been proposed to resemble small-world networks (Bettencourt et al. 2007; Yu et al. 2008; Gerhard et al. 2011). We thus wondered if networks with small RMSE were more similar to small-world networks. Optimizing the crucial parameters for low RMSE also increased the small-world-ness value *S* (see methods) of the network from *S* = 1 for uniform random networks to *S* = 1.3 for the region of best results, but there was no direct correlation: high small-world-ness values did not imply a good network fit (Fig. 4*D*). In fact, a high small-world-ness could be achieved by networks with a wide range of RMSEs. Thus, in our case, high small-world-ness values of the overall network architecture did not imply more biologically realistic structures. Notably, there was no correlation between the over all small-world-ness of the network and the reciprocity value for E → E connections.

By comparing the structure of real cortical and computer-generated networks with the same measure, we were able to identify model architectures that matched the experimentally observed connectivity statistics much better (RMSE = 0.04). However, it was unclear if the resulting network dynamics would also be comparable to those of real cortical networks. To investigate this issue, we implemented AdEx integrate-and-fire mechanisms in each cell, with parameter sets that mimic single cell dynamics in barrel cortex (see methods and Mensi et al. 2012). We set the crucial parameters (as determined in the first parameter scan) to their appropriate values and set all other, noncrucial parameters to *d* = 0, in agreement with the standard uniform random network paradigm. Synaptic weights were chosen to globally obey experimentally measured distributions (Avermann et al. 2012). The weight correlations ς_in_ and ς_out_ could be varied for each connection type individually. To achieve similar weight distributions across all neurons, synaptic weights were drawn randomly from a lognormal distribution (ς = 0). In this case the range of synaptic strengths of one neuron was equal to that of the entire population of synapses. Alternatively (ς = 1), all incoming or outgoing synapses of a given neuron were adjusted by a common scaling factor that was itself drawn independently from a log normal distribution. This led to weight distributions in which a neuron had (incoming or outgoing) synapses of similar strength so that the range of synaptic strengths onto or from one neuron was smaller than the range of strengths of the whole population (see methods). In both cases, the global weight distributions were kept equal to those measured by Avermann et al. (2012).

We investigated the stimulus response properties of the network with a protocol similar to the experiments performed in Avermann et al. (2012): We stimulated a subset of 25 excitatory neurons to evoke a single volley of synchronous spikes and recorded the postsynaptic responses in randomly chosen cells. The resulting MRH obtained over 10 iterations (with shuffled parameter sets) were set against the experimentally obtained distributions of each cell type. The similarity of the responses was quantified by calculating the negative log-likelihood ℓ of the MRH of the model, given the experimental result, and summed over all three cell types, presented as ℓ (ℓ_E_/ℓ_FS_/ℓ_NFS_), the average value followed by the cell type specific similarity values for easy evaluation. A low value of ℓ indicated a high similarity between the model response distributions and the experimental response distributions (see methods).

**Fig. 5.**
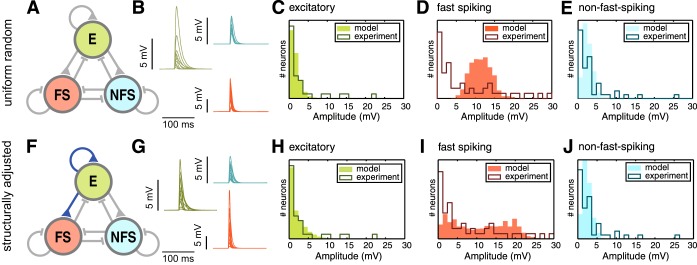
Subthreshold network response distributions *A*: network diagram as in Fig. 1. All connections are colored gray to indicate their uniform random connectivity. *B*: 10 representative voltage traces of each cell type, after stimulation with a channelrhodopsin-like stimulus (color-coded as in *A*). *C*–*E*: distributions of subthreshold network response amplitudes to a synchronous spike volley in 25 excitatory neurons, measured in excitatory (*C*), fast-spiking (*D*), and nonfast-spiking neurons (*E*). Outlined histograms are experimental results obtained by channelrhodopsin stimulation. Solid histograms show the response of a uniform random model. *F*–*J*: network diagram, voltage traces, and response distributions as in *A*–*E* for the best structurally adjusted network.

We found that the response distributions of the uniform random model [with ℓ = 154 (67/279/122)], as well as the structurally optimized model [with ℓ = 116 (64/163/120)] did not match the experimentally observed subthreshold responses (Fig. 5). Neither model could account for the long tails of the experimental MRHs, and while the structurally adjusted model fared slightly better, both models failed to produce the highly skewed response distribution of the FS population (Fig. 5, *D* and *I*). In preliminary simulations we confirmed that changing the number of stimulated cells in each trial (to account for experimental variability of the optogenetically active cell population) could not account for the observed response distributions (data not shown). We hypothesized that the mismatch between experiment and model might be due to the unadjusted structural parameters like inhibitory-inhibitory connections or stem from systematic correlations between synaptic weights originating from or terminating onto the same neuron.

The space of untested parameters that could affect the response distributions was still high (36 − 7 = 29) dimensional, so we decided to further subdivide the analysis. Since the next parameter sweep aimed to explore the subthreshold voltage responses of the network, only connections from cell types that contributed spikes to the response were taken into account here. In preliminary tests (data not shown) we found that the stimulated excitatory neurons emitted spikes but no other excitatory neurons fired. Furthermore, we found that FS neurons emitted spikes in response to the stimulus in a wide range of parameters (average 4.93 ± 3.35 spikes, with 14 spikes maximum), but NFS cells almost never spiked (average 0.08 ± 0.17 spikes with 2 spikes maximum). This meant that NFS neurons did not affect the overall dynamics of the response, and we could thus neglect the parameters of the connections to and from the NFS population for the exploration of network response distributions.

**Fig. 6.**
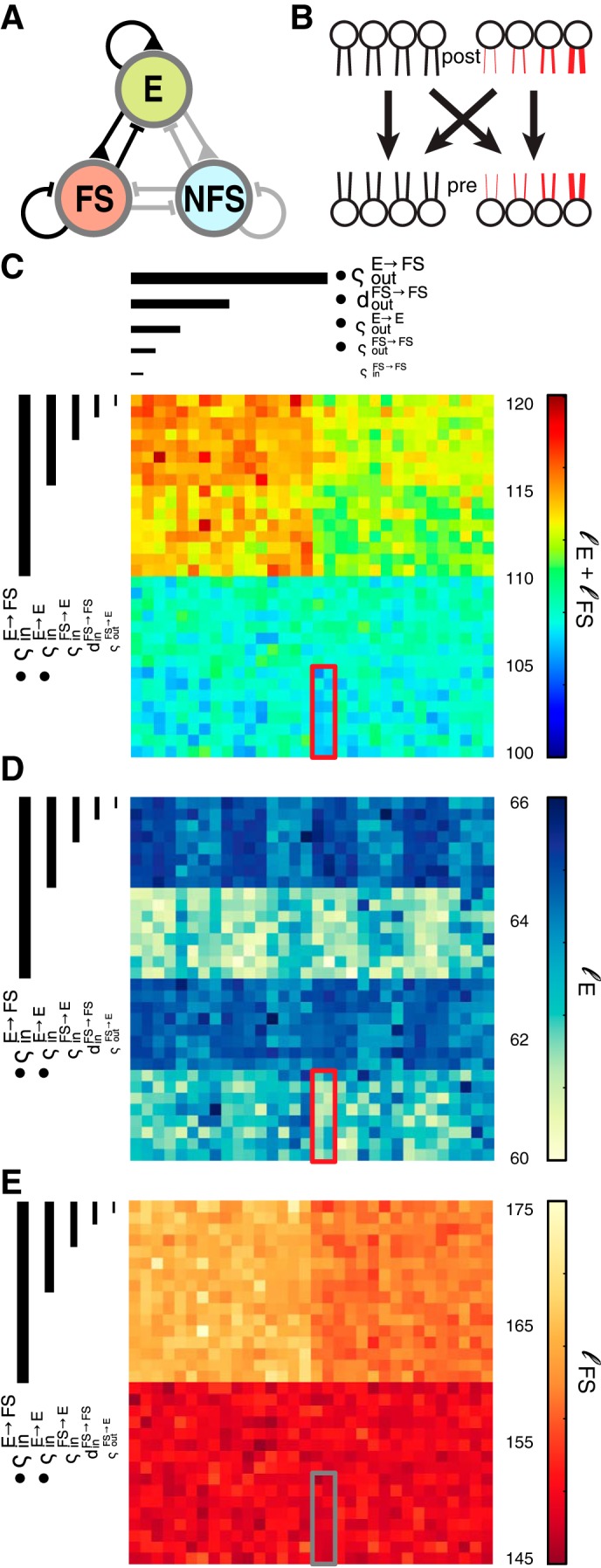
*Step II*: exploration of the dynamic responses of E and FS neurons. *A*: network diagram as in Fig. 1. Connection types that were investigated in *step II* are shown in black. *B*: Schematic of possible connectivity combinations with uniform or skewed synaptic weight correlations. *C*: similarity values ℓ of network responses to a single spike volley for every parameter combination, sorted by CBDR. The red outline shows the region of best results. *D* and *E*: similarity values ℓ_E_, ℓ_FS_, scoring only the response of the excitatory (*D*) or the fast-spiking population (*E*), with identical stacking as in *B*. Boxes indicate the overall region of best results as shown in *A*. Crucial parameters are marked with bullet points.

We also excluded the structural parameters in the FS → E connection: the inhibitory reversal potential of the excitatory cells (*V*_revI_ = −75 mV) was close to the resting membrane voltage (*V*_rest_ = −68.85 mV), so that these synapses were largely shunting, and preliminary simulations showed that their effect on maximum subthreshold responses was negligible. This exclusion step left 10 parameters, namely ς_in_^E→E^, ς_out_^E→E^, ς_in_^E→FS^, ς_out_^E→FS^, ς_in_^FS→E^, ς_out_^FS→E^, ς_in_^FS→FS^, ς_out_^FS→FS^, d_in_^FS→FS^, and d_out_^FS→FS^, to be tested (Fig. 6*A*). The cumulative similarity scores ℓ of the resulting 1,024 models (Fig. 6*C*) revealed only the parameters of the connection E → FS as crucial. For these connections, the introduction of input and output correlations (ς_in_^E→FS^ = 1 and ς_out_^E→FS^ = 1, cf. Fig. 6*C*) improved the ℓ score. When we analyzed the responses of the E and FS populations separately (Fig. 6, *D* and *E*), we uncovered additional crucial parameters. ℓ_E_, the similarity measure of only excitatory populations, increased when excitatory-excitatory connection weights were correlated (i.e., ς_in_^E→FS^ = 1) and outgoing E → E connection weights were not correlated (ς_out_^E→E^ = 0). Using ℓ_FS_ to examine only the inhibitory connections showed that *d_out_^FS→FS^ and *ς_in_^FS→FS^ out also had a substantial (although not Bonferroni-corrected significant) effect on the similarity score. Both parameters must allow uniform random network connectivity. We could thus identify an additional 6 parameters (ς_in_^E→E^, ς_out_^E→E^, ς_in_^E→FS^, ς_out_^E→E^, *ς_out_^FS→FS^, and *d_out_^FS→FS^) as crucial.

**Fig. 7.**
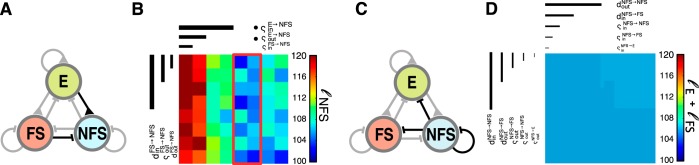
*Steps III* and *IV*: parameter scan for NFS connections. *A*: network diagram as in Fig. 1. Connection types that were investigated in *step III* are shown in black. *B*: similarity value ℓ_NFS_ of all parameter combinations of *step III* for the nonfast-spiking population, sorted with CBDR. The red outline shows the region of best results. Crucial parameters are marked with bullet points. *C*: network diagram as in Fig. 1. Connection types that were investigated in *step IV* are shown in black. *D*: similarity value ℓ of all parameter combinations of *step IV* for the nonfast-spiking population, sorted with CBDR. No crucial parameters could be identified.

In a third parameter scan, we explored the response distribution of the NFS population. We varied only the connections to the NFS population (Fig. 7*B*), while keeping the values of crucial parameters at their better value, and those of noncrucial parameters uniform random, and thus consistent with the uniform random connectivity hypothesis. In these simulations only E → NFS connectivity had an effect on the NFS population, meaning the incoming weight correlations had to be high (ς_in_^E→NFS^ = 1) and the outgoing weight correlations low (ς_out_^E→NFS^ = 0; Fig. 7).

Finally, in the last step, we constructed model networks in which all of the hitherto untested parameters were varied (Fig. 7*C*). We calculated their impact on the similarity to the experimentally observed subthreshold response distributions. We found that none had significant effects and were thus not crucial (Fig. 7*D*).

**Fig. 8.**
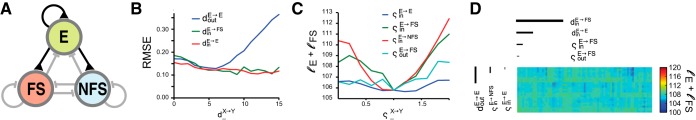
Parameter explorations for crucial, nonuniform-random parameters. *A*: network diagram. Tested connection parameters in black. *B*: RMSE for individual changes of crucial nonuniform-random structural parameters. Starting with an optimally adjusted network, each parameters is varied separately. *C*: ℓ values for individual changes of crucial nonuniform-random weight parameters. Starting with an optimally adjusted network, each parameters is varied separately. *D*: ℓ values for combined variations for all crucial nonuniform-random parameters, starting from a fully adjusted network. Each parameter was tested for 3 values (with *d* = {4, 5, 6} and ς = {0.8, 1, 1.2}). Results are sorted with CBDR.

In two additional control steps we explored the local neighborhood of all crucial, nonrandom parameters (Fig. 8). Starting with the fully adjusted network, we first tested each parameter individually for a larger range of values (Fig. 8, *B* and *C*). Similar to our initial test scans, we found that the chosen parameter values produced consistently good results for the chosen parameter values. We then performed an additional 2,187 simulations in which the crucial, nonuniform random parameters were varied between three values (d_in_, d_out_ = {4, 5, 6}, and ς_in_, ς_out_ = {0.8, 1, 1.2}), but similarity values did not improve substantially for any combination of parameters (Fig. 8*D*).

**Fig. 9.**
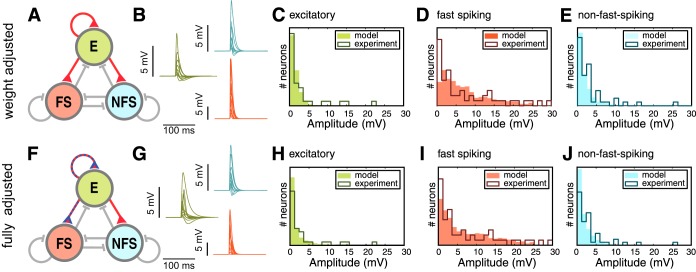
Network response distributions II: weight- and fully adjusted architectures. *A*: network diagram. The network was setup with all connections set to uniform random structure but crucial weight parameters were adjusted as required. The connections in red had at least 1 crucial and skewed parameter, connections in gray were constructed using the uniform random setup. *B*: 10 representative voltage traces of each cell type, after stimulation with a channelrhodopsin-like stimulus (color coded as in *A*). *C*–*E*: distribution of response amplitudes in excitatory (E; *B*), fast-spiking (FS; *C*), and nonfast-spiking neurons (NFS; *D*) following a synchronous spike volley in 25 excitatory neurons. Outlines are experimental results while solid bars show the model predictions. *F*–*J* like *A*–*E* but with optimal structurally and weight adjusted connection parameters, as indicated by the blue and red colored connections in *F*.

Although the 36-dimensional parameter space we explored remained vastly undersampled (in fact, we only tested 4.5 * 10^−6^% of all possible parameter combinations), we were able to find a parameter combination that produced substantially better results than the random uniform network. The response distributions of the fully adjusted network (Fig. 9, *F*–*J*) show a higher similarity to the biologically measured MRH with an average negative log-likelihood value of 106 (63/151/102) [compare with 154 (67/279/122) for uniform random networks; Fig. 5] and visually a much better fit as well, indicating a more plausible network architecture for stereotypically generated networks of layer 2/3. These structurally and weight adjusted networks also performed better than networks that were only adjusted in the synaptic weight correlations [ℓ = 111 (67/163/104); Fig. 9, *A*–*E*], indicating that both structure and weight statistics must be altered from the initially assumed uniform random structure.

## DISCUSSION

Our results confirm that uniform random networks are not able to accommodate the connectivity patterns found in biological experiments (Yoshimura and Callaway 2005; Yoshimura et al. 2005). To bring a network model into better agreement with these datasets, it was necessary to change the structure of specific in- and out-degree distributions (*d* = 5 for d_in_^E→E^, d_in_^E→FS^, and d_out_^E→E^) so that these connection types became heterogeneous in the number of connections per neuron. We found that other connection parameters (d_in_^NFS→E^, d_out_^E→FS^, d_out_^E→NFS^, *d_in_^E→NFS^, and *d_out_^FS→FS^) also affected the similarity measure between model and experimental data significantly, but those had to remain at the value (*d* = 0) that guaranteed uniform random connectivity.

Neither networks with uniform random connectivity nor the structurally adjusted architecture could reproduce the dynamic responses to a stimulus protocol similar to in vitro channelrhodopsin stimulation experiments published in the recent past (Avermann et al. 2012). To reconcile the network's dynamical behavior with those experiments, we had to further adjust local weight correlations ς. These manipulations changed the weight of existing synapses cell-wise by a common factor, without affecting the global weight statistics or the connectivity structure. We found highly cell-type specific connectivity requirements: all excitatory connections required correlations in the input weights (ς = 1 for ς_in_^E→E^, ς_in_^E→FS^, and ς_in_^E→NFS^), and the excitatory output weights onto fast-spiking neurons (ς_out_^E→FS^) also had to be correlated cellwise to match biological measurements. Additionally, we found parameters that needed to remain at the value for uniform random networks (ς = 0 for ς_out_^E→E^, ς_out_^E→NFS^, and *ς_out_^FS→FS^).

The crucial parameters that affected network dynamics as described here are almost exclusively connections from the excitatory population to other cell populations, and two of the other parameters, *d_out_^FS→FS^ and *ς_out_^FS→FS^ (in addition to *d_in_^E→NFS^, which alters synapse distributions that originate from E neurons) are weakly significant, i.e., they fail the more conservative Bonferroni-corrected significance levels. This may hint at a systematic error in our dataset, likely due to the stimulus we used. The synchronous volley of spikes in the excitatory population in an otherwise silent network may have had a larger impact on excitatory connections because it naturally favored excitatory activity. Any other, nonexcitatory contribution to the poststimulus response can be understood as a disynaptic echo of the original stimulus. By design of the experiment, such echoes are less likely to have a significant impact on the observed dynamics. In the future it may become possible to include data in which a similar channelrhodopsin stimulus is delivered to the FS or NFS neurons, or in a perpetually active, in vivo or in vivo-like network state. In such scenarios, the wiring requirements for inhibitory neurons would likely become more prominent.

The applied changes to the network architecture are conceptually simple because we aimed to find a minimum number of stereotypical rules that allow easy but accurate reconstruction of layer 2/3 network models. The architecture of biological networks is likely more complex than the networks built here and may not be achievable with our purely stochastic methods of assigning connections. However, the proposed parameter changes offer insight into which connections parameters are affecting network architecture and behavior in a way that significantly alters their similarity to experimentally observed facts and guide the search for plasticity rules that ultimately govern the creation of architecture in real networks.

Although the crucial connection parameters should be taken into account when building simplified models, it should be noted that the parameters we introduced here do not necessarily create the correct, realistic network architectures. We merely searched for the best response range to a certain search paradigm (Prinz 2010). For example, our results indicate that an in-degree distribution with high variance for the E → FS connections leads to a network with high similarity to experimental measures. This suggests the existence of highly connected (hub neurons) but also of many FS cells with only sparse excitatory input. Such sparsely connected interneurons have not been shown to exist in biology. In fact, recent results show that FS neurons are most often highly connected with locally very dense excitatory input (Hofer et al. 2011; Packer and Yuste 2011). One interpretation of our results would be that the high variance of E → FS does not create high spatial connectivity directly but rather as an approximation without the explicit spatial scale with highly connected FS neurons necessary for the dynamics. The sparsely connected neurons that are also created by this parameter on the other hand, have no impact on the dynamics, and can thus be neglected. In fact, pruning the sparse connections of FS neurons, we find that the resulting MRHs and similarity measure ℓ in our network do not change (data not shown).

The accuracy of our results necessarily hinges on the fidelity with which the experimental data reflect reality. We have neglected potential slicing artifacts from injury, as well as the possibility that experimental choices and biases, e.g., the nature of the stimulus (a single excitatory spike volley), may disadvantage the accuracy of certain connection type statistics or even conceal their involvement in the recorded phenomenon. It should also be noted that we combined experimental data from different species and brain regions, namely layer 2/3 rat visual cortex (Yoshimura and Callaway 2005; Yoshimura et al. 2005) for structural adjustments and layer 2/3 mouse barrel cortex (Avermann et al. 2012) to explore the synaptic weights. There is no a priori reason to assume that these sensory cortices must obey the same architectural rules. The good agreement of reciprocity values obtained from layer 5 of visual (Song et al. 2005) and somatosensory (Perin et al. 2011) cortexes in the rat with our best network models may hint at the existence of a universal, canonical cortical microcircuit, but a final verdict is not in sight.

Regardless of the universality of our results, we can say with certainty that the uniform random connection hypothesis fails to accommodate the specific datasets we tested. Moreover, we can find a better solution that obeys the experimental restrictions and produces similar statistics as observed in the data as it stands at this point in time. With ever-increasing computational might of the coming decades, it will become necessary, desirable, and, importantly, possible to repeat and refine the presented analysis with new and more complete datasets.

In summary, we show here that even conceptually simple experiments that do not aim at revealing network connectivity like the channelrhodopsin stimulation in Avermann et al. (2012) can be used to investigate network architecture in a meaningful way. With the help of computationally inexpensive extensions we adjusted the architecture of uniform random networks to reflect biological networks more accurately and thus provide a framework to construct biological plausible networks that may help to improve the conclusions of theoretical models in the future. We provide these new and more plausible network architectures as example files and the code to construct them online at ModelDB (http://senselab.med.yale.edu/modeldb/ShowModel.asp?model=156040).

## GRANTS

This research was supported by Swiss National Science Foundation Grant No. 20002013287 (Coding Characteristics), ERC Grant No. 268689 (MultiRules), and CRSIK0 122697 (Sinergia). Additionally, C. Tomm was funded by European Union Framework 7 ICT Project 215910 (BIOTACT), and T. P. Vogels was supported by the European Communitys Seventh Framework Marie Curie International Reintegration Grant No. 268436 (Cifine) and a Sir Henry Dale Wellcome Trust and Royal Society Research Fellowship (WT100000).

## DISCLOSURES

No conflicts of interest, financial or otherwise, are declared by the author(s).

## AUTHOR CONTRIBUTIONS

C.T., W.G., and T.P.V. conception and design of research; C.T. and M.A. performed experiments; C.T. and M.A. analyzed data; C.T., C.P., W.G., and T.P.V. interpreted results of experiments; C.T. and T.P.V. prepared figures; C.T. and T.P.V. drafted manuscript; C.T., M.A., C.P., W.G., and T.P.V. edited and revised manuscript; C.T., M.A., C.P., W.G., and T.P.V. approved final version of manuscript.
